# Phosphorylated Neurofilament Heavy Chain in Cerebrospinal Fluid and Serum as a Biomarker of Axonal Injury in Algerian Patients with Multiple Sclerosis

**DOI:** 10.3390/diseases14080266

**Published:** 2026-07-24

**Authors:** Bouchra Nour El Houda Baiski, Zoulikha Mokrani, Sara Mimi Atmani, Fatma Zohra Ider, Nabila Lakri, Fatma Zohra Souid, Samia Chaib, Assia Galleze

**Affiliations:** 1 Laboratory of Cellular and Molecular Biology, Faculty of Biological Sciences, Houari Boumediene University of Sciences and Technology (USTHB), Algiers 16111, Algeriasaramimi.atmani_fsb@usthb.edu.dz (S.M.A.);; 2 Immunology Department, Mohamed Seghir Nekkache Hospital, Algiers 16000, Algeria; 3 Department of Biology and Organism Physiology, Faculty of Biological Sciences, Houari Boumediene University of Sciences and Technology (USTHB), Algiers 16111, Algeria; mokranizoulikha@yahoo.fr; 4 Neurology Department, Mohamed Seghir Nekkache Hospital, Algiers 16000, Algeria

**Keywords:** multiple sclerosis, pNF-H, cerebrospinal fluid, axonal injury, biomarkers, neurodegeneration

## Abstract

Objective: Axonal injury is a key determinant in irreversible disability in multiple sclerosis (MS). Reliable biomarkers of neuroaxonal damage are essential for improving diagnosis, monitoring disease progression, and evaluating treatment response. This study investigated the relationship between phosphorylated neurofilament heavy chain (pNF-H), clinical characteristics, and conventional cerebrospinal fluid (CSF) and serum biomarkers in Algerian patients with MS. Methods: A total of 102 participants were enrolled. Clinical, immunological, and biochemical parameters were assessed, including the Expanded Disability Status Scale (EDSS), oligoclonal bands (OCBs), IgG index, albumin quotient, and pNF-H concentrations in paired CSF and serum samples. Results: OCBs were detected in 85.5% of patients, and 65.21% exhibited intrathecal immunoglobulin synthesis, with a median IgG index of 0.87. Patients with progressive MS were significantly older and more disabled than those with relapsing–remitting MS (age: *p* = 0.01; EDSS: *p* = 0.0007). OCB-positive patients had significantly higher IgG index values (*p* = 0.0004), but OCB status was not associated with age, EDSS, or albumin quotient. EDSS correlated positively with age (*p* = 0.0007), albumin quotient (*p* = 0.01), and IgG index (*p* = 0.001). Both CSF and serum pNF-H levels were significantly elevated in MS patients compared with NSDs group (*p* < 0.001). Increased pNF-H concentrations were associated with progressive disease and greater disability (EDSS ≥ 5). Conclusions: Elevated pNF-H levels in CSF and serum are associated with disease severity and progressive MS, supporting their potential as complementary biomarkers of neuroaxonal damage and clinical disability in routine MS assessment.

## 1. Introduction

Multiple sclerosis (MS) is a chronic immune-mediated disorder of the central nervous system characterized by inflammation, demyelination, and progressive axonal injury [1,2,3,4,5]. Axonal loss is a fundamental pathological substrate underlying irreversible neurological decline, with additional contributions from synaptic dysfunction and degeneration [6,7]. This process occurs throughout the disease course via two main mechanisms: acute axonal transection within inflammatory lesions and delayed neurodegeneration following prior injury, when compensatory mechanisms fail. This latter represents a major determinant of cumulative disability [8,9,10,11,12]. Despite its clinical relevance, sensitive and reliable outcome measures for assessing neuroprotective strategies targeting delayed axonal loss remain limited. Conventional clinical endpoints lack the sensitivity to detect early disease worsening [13].

Neurofilaments (NFs) are major structural components of neurons, predominantly located in axons. NFs are heteropolymers composed of three major isoforms: light (NfL), medium (NfM), and heavy (NfH) neurofilaments, classified according to their molecular weight. Each subunit consists of an N-terminal head domain, a conserved α-helical rod domain, and a variable C-terminal tail domain [14].

NFs are primarily responsible for maintaining the mechanical integrity of neurons. Their filamentous network provides structural support to long axons, enabling resistance to mechanical stress and adaptation to environmental challenges. Furthermore, NFs serve as scaffolding for the spatial organization and intracellular transport of organelles, including mitochondria and the endoplasmic reticulum, and contribute to the stabilization of microtubule networks [15,16]. NFs have been shown to be a promising biomarker across a spectrum of neurodegenerative diseases, including multiple sclerosis, Alzheimer’s disease, frontotemporal dementia, amyotrophic lateral sclerosis, and Parkinsonian disorders [17,18,19,20,21]. Neuroaxonal injury, such as that observed in multiple sclerosis, leads to the release of NFs into the CSF and, depending on the severity of the damage, into the peripheral circulation. Due to their neuronal specificity and abundance, NFs are considered promising biomarkers of axonal injury [22,23,24,25,26,27].

This study aimed to evaluate pNF-H levels in CSF and serum as a biomarker of axonal injury in Algerian patients with MS and to explore its association with clinical parameters of the disease and disability as measured by the Expanded Disability Status Scale (EDSS).

## 2. Materials and Methods

### 2.1. Study Subjects

A total of 102 participants were enrolled in this study from Mohamed Seghir Nekkache Hospital, Algiers, between March 2024 and February 2026, including 69 patients with multiple sclerosis and 33 patients with other neurological disorders. Inclusion criteria comprised patients with retrobulbar optic neuritis, motor or sensory disturbances, white matter abnormalities on cerebral magnetic resonance imaging, and myelitis on spinal magnetic resonance imaging according to McDonald criteria for the diagnosis of multiple sclerosis. Exclusion criteria encompassed the presence of other neurological or neurodegenerative disorders; history of acute or chronic systemic inflammatory or autoimmune diseases other than multiple sclerosis; signs of central nervous system infections; presence of a malignant condition or serious systemic disease; recent head trauma or spinal cord injury; and immunosuppressive or corticosteroid therapy within a defined period prior to sample collection. This study has been approved by the local ethics committee, the Algerian National Agency for Research in Health Sciences (ATRSS ex-ANDRS), in compliance with the Helsinki declaration (code number 58-DFPR-ATRSS-AAP-2018 and approved date 29 April 2018).

### 2.2. Quantitative Measurement of Albumin and IgG

Quantitative measurements of albumin and IgG were performed in 102 paired serum and CSF using a Siemens BN ProSpec nephelometric system (Siemens Healthcare Diagnostics, Marburg, Germany), according to the manufacturer’s instructions.

### 2.3. Evaluation of Intrathecal IgG Synthesis Using the Reiber Diagram

Intrathecal IgG synthesis was assessed in 102 paired CSF and serum samples using the Reiber diagram, a quantitative method that distinguishes intrathecal IgG production from passive serum diffusion across the blood–CSF barrier. Albumin and IgG concentrations were measured in paired CSF and serum samples using standard immunochemical assays (BN ProSpec System, Siemens Healthineers, Erlangen, Germany). The CSF/serum albumin quotient (QAlb = AlbuminCSF/Albuminserum) and IgG quotient (QIgG = IgGCSF/IgGserum) were calculated. The measured QIgG was then compared with the theoretical upper reference limit (QLim), calculated as a function of QAlb according to the Reiber hyperbolic function. The results were plotted on a logarithmic Reiber diagram. Samples with QIgG values above the QLim curve were considered positive for intrathecal IgG synthesis, whereas values below the QLim curve indicated normal passive transfer of IgG across an intact blood–CSF barrier. In addition, the IgG Index was calculated as the ratio of the IgG quotient (QIgG) to the albumin quotient (QAlb). An IgG Index > 0.70 was considered indicative of intrathecal IgG synthesis.

### 2.4. Determination of Oligoclonal Bands

Oligoclonal bands (OCBs) were analyzed in paired CSF and serum samples obtained from 102 participants using the SAS-3 analyzer with Helena HL-3-0362SA kits (Helena Biosciences Europe, Gateshead, UK). The analysis consisted of isoelectric focusing on agarose gel, followed by immunoblotting with IgG-specific antibodies, in a semi-automated procedure. The OCB patterns were assessed according to international guidelines, and samples were categorized as “with CSF-specific OCBs” or “without CSF-specific OCBs.” OCBs were considered as CSF-specific when two or more additional bands were present in the CSF compared to the corresponding serum sample.

### 2.5. sNFH Measurements

CSF and serum concentrations of pNF-H were quantified in paired samples obtained from 102 participants using a commercially available sandwich enzyme-linked immunosorbent assay (ELISA) kit (Human pNF-H ELISA Kit, Catalog No. EEL191, Thermo Fisher Scientific, Waltham, MA, USA), according to the manufacturer’s instructions. Prior to analysis, CSF and serum samples were centrifuged to remove cellular debris, aliquoted, and stored at −80 °C until use. Samples were thawed only once, mixed gently without vortexing, and repeated freeze–thaw cycles were avoided. All reagents were allowed to equilibrate to room temperature before use. A standard curve was prepared for each assay by serial dilution of the supplied pNF-H standard to obtain concentrations of 5000, 2500, 1250, 625, 312.5, 156.25, 78.13, and 0 pg/mL. Standards and samples (100 μL/well) were added to microplate wells pre-coated with a monoclonal antibody specific for human pNF-H and incubated for 90 min at 37 °C. After aspiration of the reaction mixture, 100 μL of biotinylated anti-pNF-H detection antibody was added to each well and incubated for 60 min at 37 °C. Wells were then washed three times with 1× wash buffer before incubation with 100 μL of horseradish peroxidase (HRP)-conjugated streptavidin for 30 min at 37 °C. Following five additional washing steps, 90 μL of tetramethylbenzidine (TMB) substrate solution was added and the plate was incubated for approximately 15 min at 37 °C in the dark. The enzymatic reaction was terminated by adding 50 μL of stop solution (1 M sulfuric acid), and absorbance was measured at 450 nm within 10 min using a microplate reader. A standard calibration curve was generated for each plate and pNF-H concentrations were calculated by interpolation from the standard curve and expressed as pg/mL. Samples with absorbance values exceeding the upper limit of the standard curve were diluted appropriately and reanalyzed. The analytical measuring range of the assay was 78.13–5000 pg/mL, with an analytical sensitivity of 46.88 pg/mL. According to the manufacturer, the intra-assay coefficient of variation ranged from 3.54% to 6.63%, while the inter-assay coefficient of variation ranged from 4.19% to 6.42%. The assay specifically detects human phosphorylated neurofilament heavy chain (pNF-H).

### 2.6. Statistical Analysis

Statistical analyses were performed using GraphPad Prism version 8.0 (GraphPad Software, San Diego, CA, USA). Comparisons between two independent groups were conducted using the Mann–Whitney U test, whereas comparisons among three or more groups were performed using the Kruskal–Wallis test. When the Kruskal–Wallis test revealed a statistically significant difference, pairwise comparisons were carried out using Dunn’s post hoc test with Bonferroni correction to account for multiple comparisons. Correlations between variables were assessed using Spearman’s rank correlation coefficient. All statistical tests were two-sided, and a *p*-value < 0.05 was considered statistically significant.

## 3. Results

Clinical data of patients are presented in Table 1. All patients were enrolled after the diagnosis of multiple sclerosis had been confirmed according to the 2017 revised McDonald criteria. Cerebrospinal fluid and serum samples were collected at the time of the routine diagnostic evaluation, and all patients were in the relapse phase at the time of sampling. Patients were divided into two groups. The first group comprised 69 subjects with MS (31 men and 38 women), a sex ratio of 1.22 and the second, 33 subjects with nervous system disorders (22 men and 11 women), a sex ratio of 2.0. The median age of patients was 30.0 [23.5–40.0] and 43.5 [29.25–54.75], respectively. MRI findings demonstrated supratentorial and infratentorial demyelinating lesions, with spinal cord involvement in many cases. Several MRI reports described active contrast-enhancing lesions, indicating ongoing inflammatory disease activity. The cohort was predominantly composed of relapsing–remitting multiple sclerosis patients (76.8%), followed by progressive forms (PMS) (23.18%). The median EDSS score was 2.0 [1.25–3.25]. Oligoclonal bands (OCBs) were detected in 59 patients (85.5%). Among patients with available Reiber classification, Zone 4 was the most frequent pattern (57.1%). According to Reiber diagram interpretation, 45 patients (65.21%) demonstrated intrathecal IgG synthesis. Separately, the median IgG index was 0.87 (IQR 0.64–1.17), and 47 patients (68.11%) had an elevated IgG index (>0.7).

When comparing RRMS patients with progressive forms PMS, patients with progressive MS were significantly older (median 35.0 vs. 28.0 years, *p* = 0.01) and had higher disability scores (median EDSS 5.5 vs. 2.0, *p* = 0.0007). No significant differences were observed between the groups regarding albumin quotient (*p* = 1.00), IgG index (*p* = 0.12), or OCBs count (*p* = 0.30).

Patients with positive oligoclonal bands had significantly higher IgG index values compared to OCBs-negative patients (*p* = 0.0004). However, our results did not show any association between the OCB positivity with the EDSS (*p* = 0.16), albumin quotient (*p* = 0.29), or age (*p* = 0.26).

Moreover, Spearman correlation analysis demonstrated that the EDSS was positively correlated with age (*p* = 0.0007), albumin quotient (*p* = 0.01), and IgG index (*p* = 0.001), while our findings did not reveal any correlation between the EDSS and the OCB count (*p* = 0.87) (Table 2).

**Figure 1:**
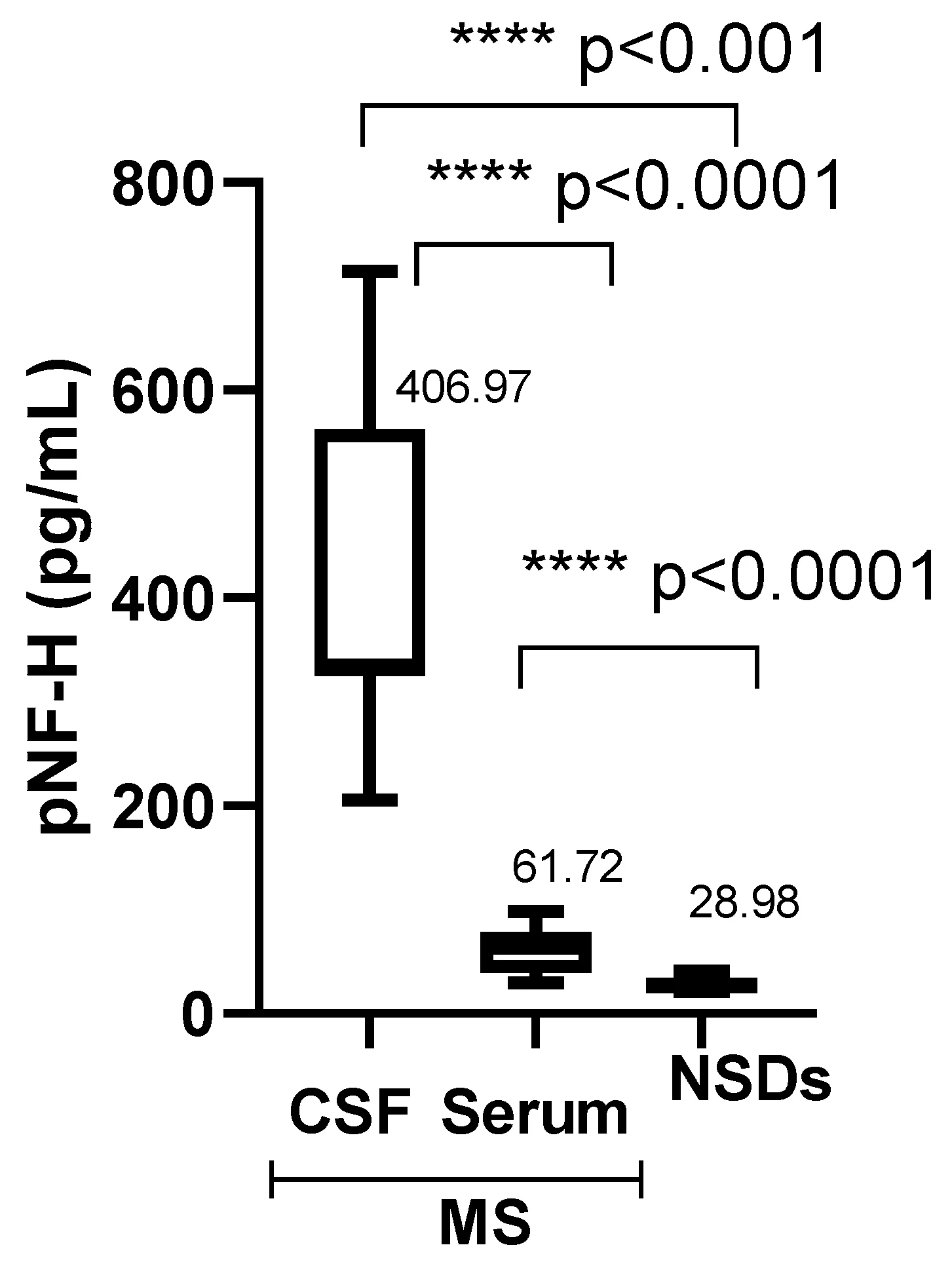
pNF-H concentrations in CSF and serum of patients with MS (CSF, *n* = 69; serum, *n* = 69) and in patients with NSDs (*n* = 33). Data are presented as box-and-whisker plots, in which the center line represents the median, the box represents the interquartile range (IQR; 25th–75th percentiles), and the whiskers indicate the minimum and maximum values. Comparisons among groups were performed using the Kruskal–Wallis test, followed by Dunn’s post hoc test with Bonferroni correction for multiple comparisons. All tests were two-sided, and a *p* value < 0.05 was considered statistically significant, **** *p* < 0.0001.

**Figure 2:**
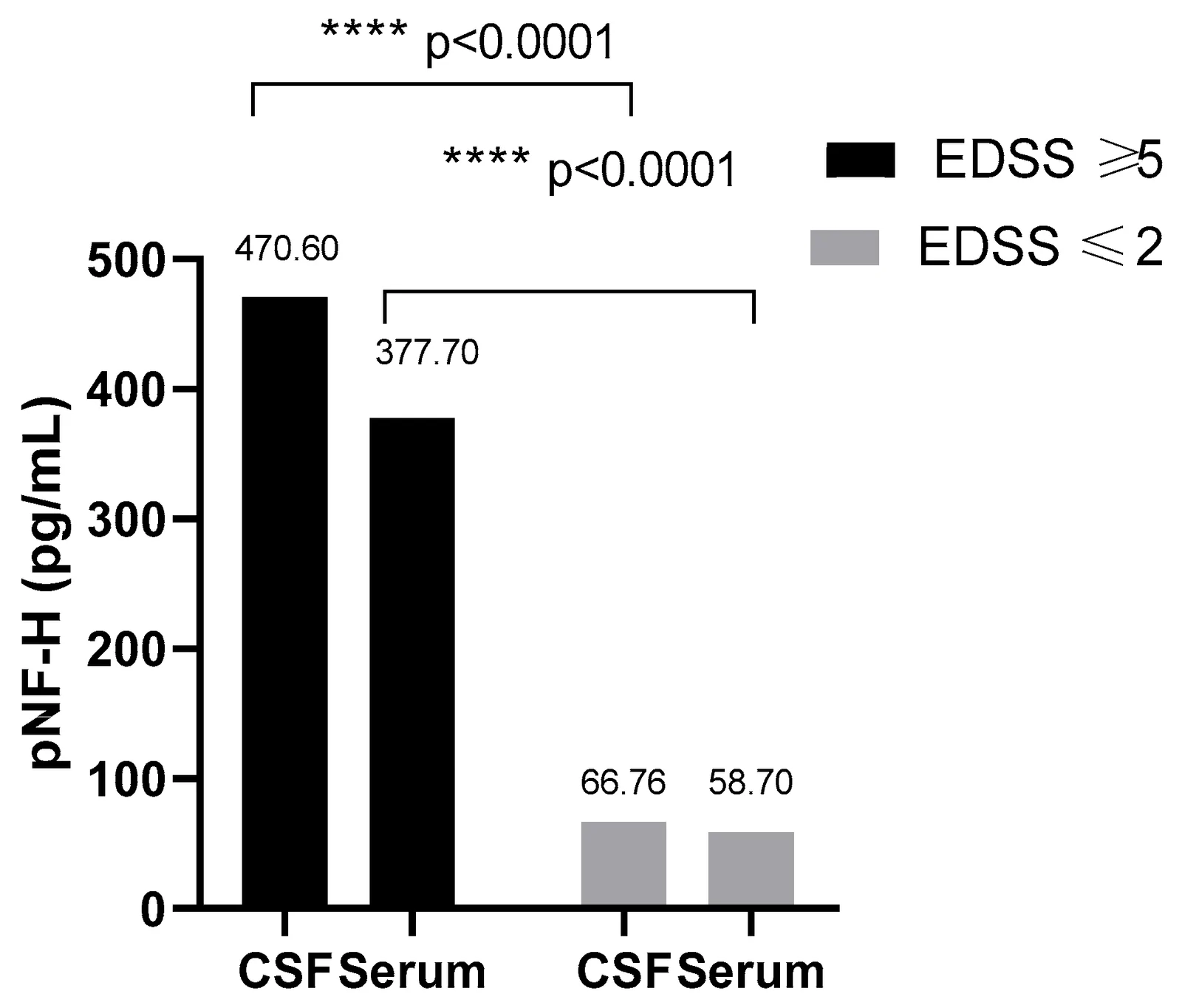
CSF and serum pNF-H concentrations in MS patients with EDSS scores ≥ 5 (*n* = 25) and EDSS scores ≤ 2 (*n* = 44). Bars represent the median pNF-H concentrations, and error bars represent the interquartile range. Comparisons were performed using the Mann–Whitney U test. All tests were two-sided, and a *p*-value < 0.05 was considered statistically significant, **** *p* < 0.0001.

**Figure 3:**
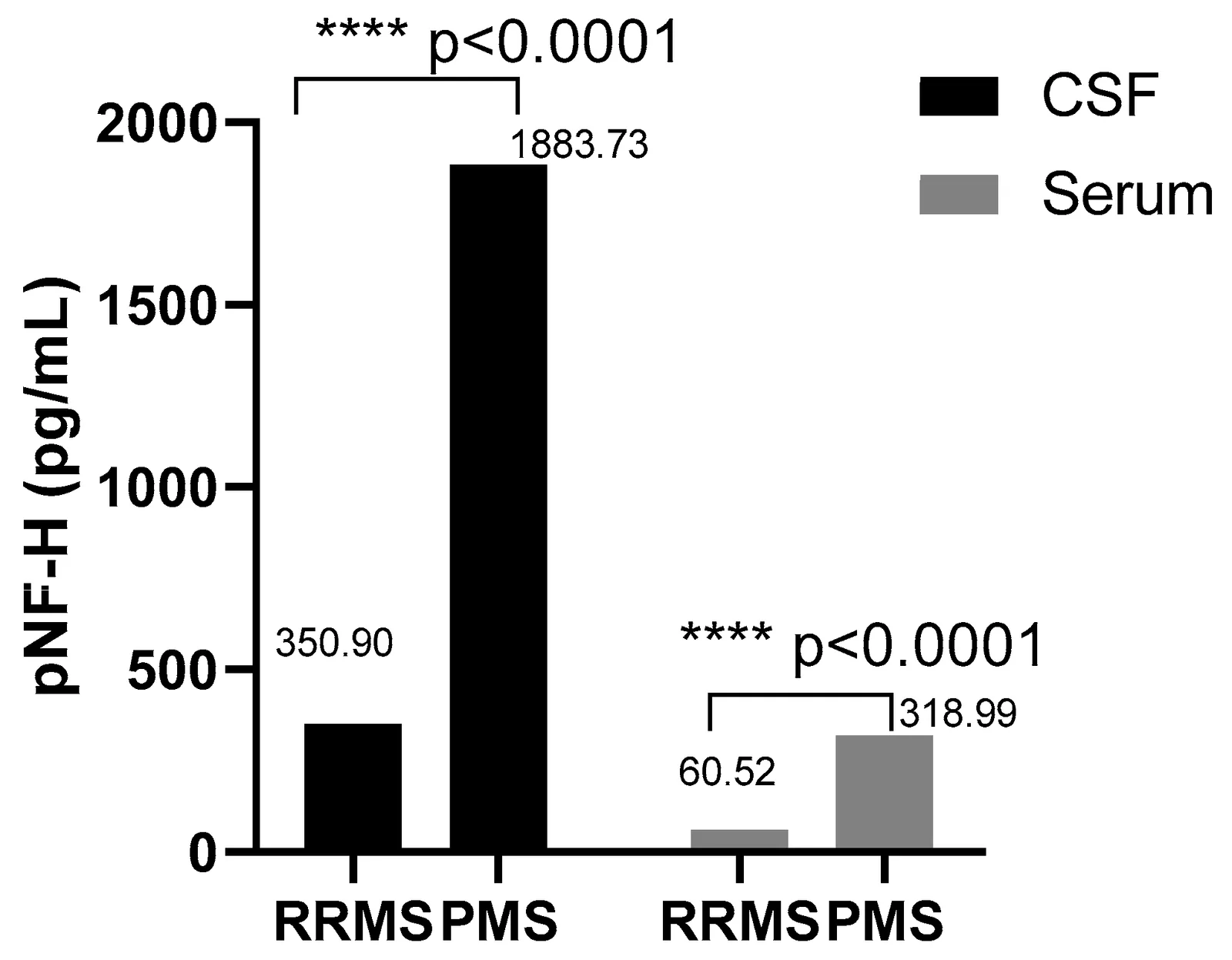
Comparison of pNF-H concentrations in CSF and serum between patients with RRMS (*n* = 53) and PMS (*n* =16). Data are presented as median with interquartile range (IQR). Comparisons between RRMS and PMS were performed using the Mann–Whitney U test. All statistical tests were two-sided, and a *p*-value < 0.05 was considered statistically significant, **** *p* < 0.0001.

Our results indicated that CSF and serum pNF-H concentrations were significantly higher in MS patients compared to the NSD group (*p* < 0.001) (Figure 1). Moreover, elevated levels of pNF-H were primarily associated with higher disability scores (EDSS ≥ 5) and PMS phenotypes (SP, PP and PR), whereas lower levels were typically observed in patients with minimal disability (EDSS ≤ 2) (Figure 2 and Figure 3).

**Figure 4:**
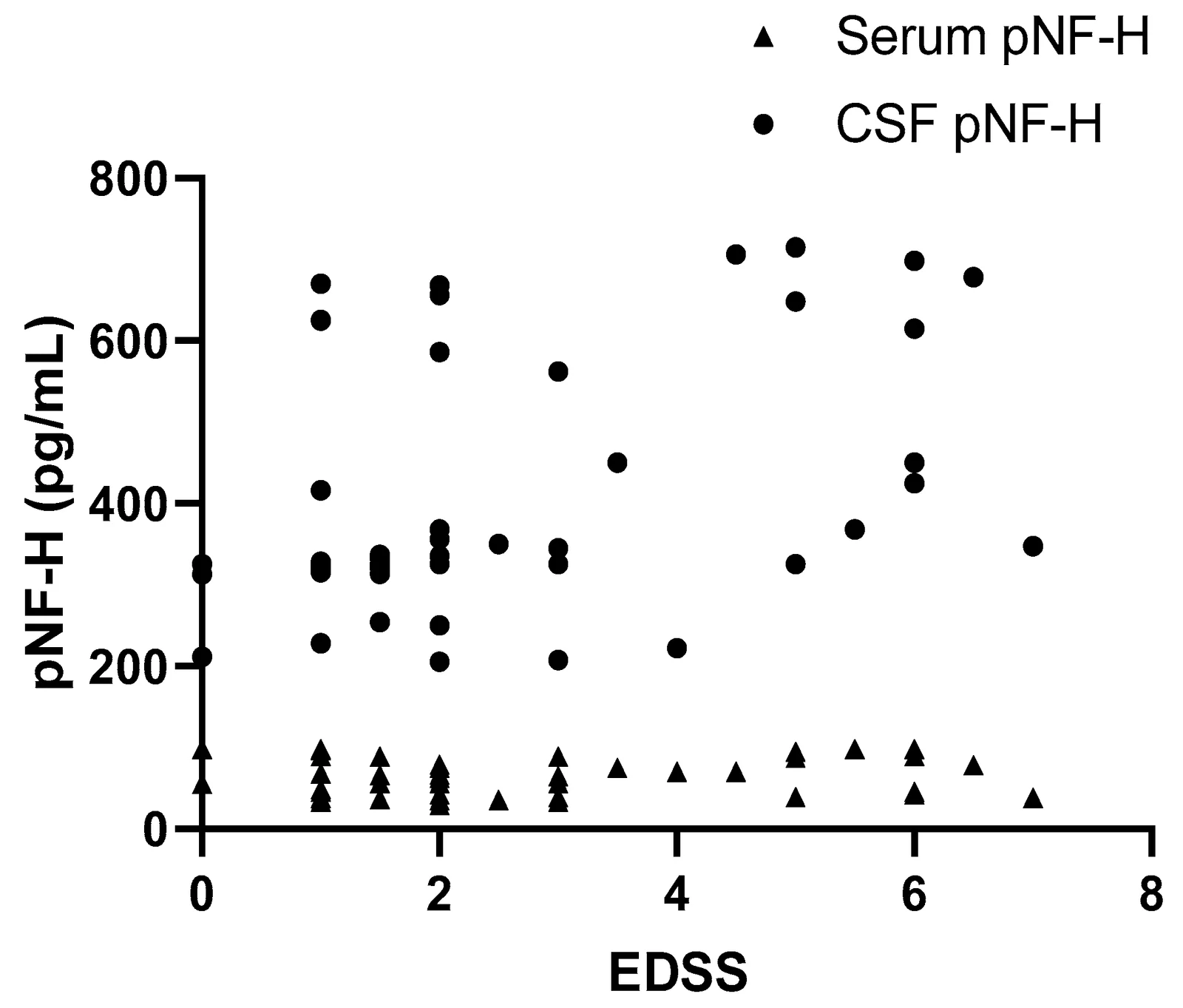
Relationship between the EDSS score and pNF-H concentrations in CSF (*n* = 69, ●) and serum (*n* = 69, ▲) of patients with MS. Correlations were assessed using Spearman’s rank correlation analysis.

Spearman’s correlation analysis revealed that EDSS scores were positively associated with pNF-H concentrations in both biological compartments. CSF pNF-H exhibited a moderate positive correlation with EDSS (ρ = 0.38, *p* = 0.005), whereas serum pNF-H demonstrated a weaker but statistically significant correlation (ρ = 0.16, *p* = 0.02) (Figure 4). The stronger association observed in CSF suggests that CSF pNF-H more accurately reflects the extent of axonal injury and neurological disability than serum pNF-H. These findings support the potential utility of CSF pNF-H as a biomarker of disease severity in multiple sclerosis, while serum pNF-H, although significantly associated with disability, appears to provide a less robust measure of neuroaxonal damage.

**Figure 5:**
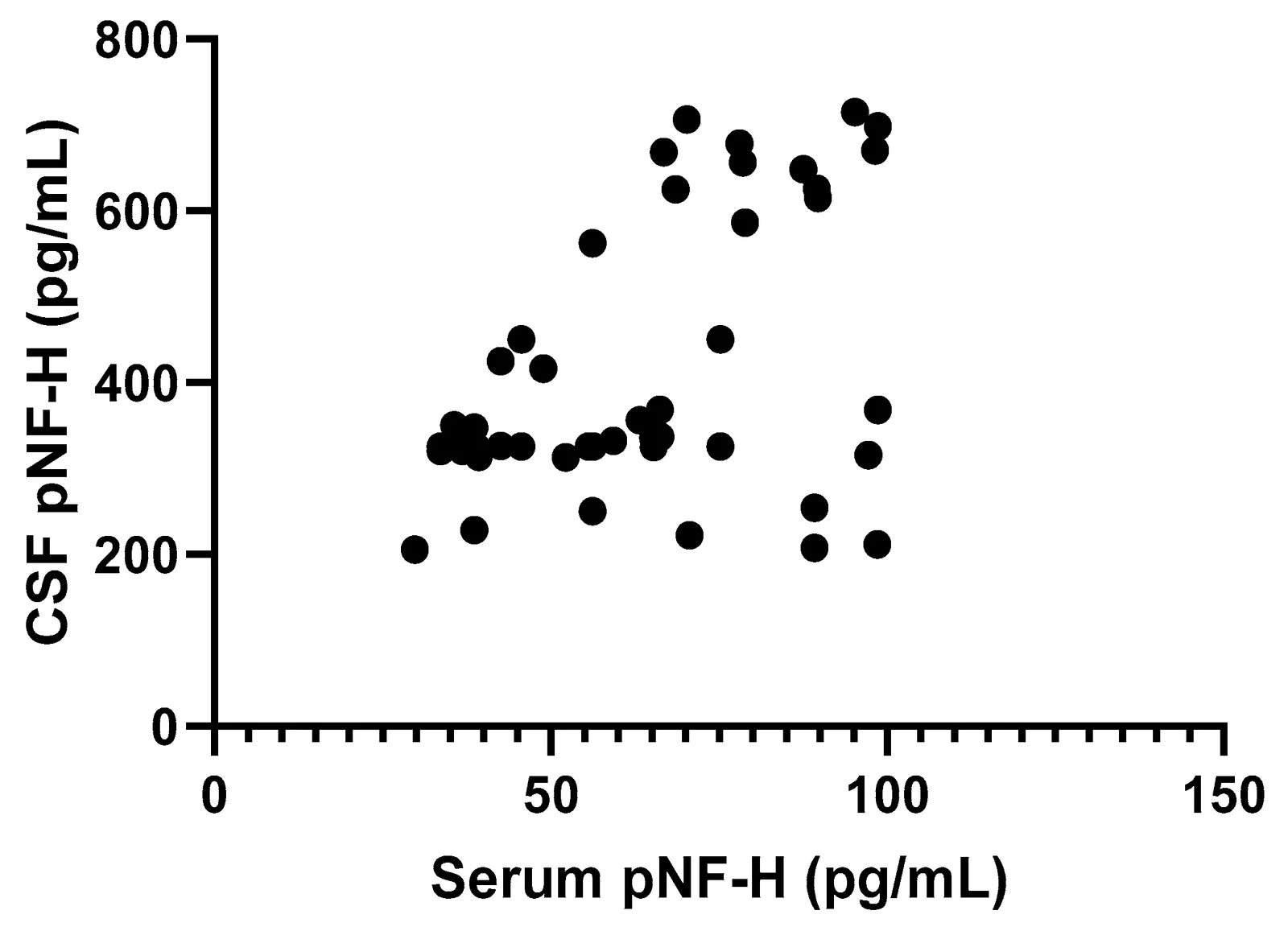
Correlation between pNF-H concentrations in serum and CSF in patients with MS (*n* = 69). The association between serum and CSF pNF-H concentrations was assessed using Spearman’s rank correlation analysis.

The relationship between serum and CSF pNF-H concentrations was assessed. A significant positive correlation was observed between serum and CSF pNF-H levels (ρ = 0.47, *p* = 0.0006), indicating that higher serum concentrations were associated with higher CSF concentrations (Figure 5).

## 4. Discussion

In this study, we evaluated serum and CSF immunological markers in a cohort of patients with multiple sclerosis and explored their association with clinical disability. Our findings confirm the predominance of intrathecal humoral immune activation, as demonstrated by the high frequency of oligoclonal band positivity, the elevated IgG index, and Reiber plot profiles consistent with intrathecal synthesis.

The high prevalence of OCB positivity (85.5%) in our cohort is consistent with previous studies in which rates of 85–95% in patients with clinically definite MS have been reported. OCBs are immunoglobulin banding patterns that reflect intrathecal IgG production and are considered markers of humoral immune activity within the central nervous system (CNS) in MS. Their presence reflects persistent, compartmentalized inflammation within the CNS rather than passive diffusion from peripheral blood [28,29]. In our study, the results indicated that the OCB positivity was strongly associated with a higher IgG index, reinforcing the internal consistency of CSF markers of intrathecal immunoglobulin synthesis.

The predominance of Reiber zone 4 (intrathecal synthesis without blood-CSF barrier dysfunction) further supports the concept that MS is primarily characterized by local immune activation rather than systemic leakage. This finding is consistent with previous work by Reiber et al., who have demonstrated that most MS patients exhibit intrathecal IgG production in the absence of significant blood-CSF barrier disruption [30]. More recent studies have confirmed that intrathecal IgG production remains a hallmark of neuroinflammatory processes in multiple sclerosis and can occur even in the presence of normal or mildly altered blood-CSF barrier function [31]. However, a subgroup of patients in our cohort belonged to Reiber zone 3, indicating concomitant BBB dysfunction. This subgroup could correspond to patients with higher inflammatory activity or more active disease phases.

Most interestingly, our findings revealed a significant correlation between the IgG index and disability (EDSS), suggesting that increased intrathecal immunoglobulin synthesis may be associated with greater disease severity. This observation is partially supported by recent studies indicating that intrathecal IgG production reflects ongoing neuroinflammatory activity; however, its relationship with clinical disability remains heterogeneous. While some studies have reported limited or no direct correlation between CSF immunological markers and disability progression, others have demonstrated that increased intrathecal immunoglobulin synthesis is associated with neuroaxonal damage and may contribute to long-term disability progression [32].

Furthermore, our results demonstrated a significant positive correlation between the albumin quotient (Alb_Q) and disability (EDSS), suggesting that BBB dysfunction may contribute to clinical impairment. Although BBB disruption is not the primary pathological driver of multiple sclerosis, it facilitates immune cell infiltration into the central nervous system and amplifies neuroinflammatory processes. Recent evidence indicates that elevated QAlb values are associated with increased inflammatory activity and neuroaxonal damage, potentially reflecting a more severe disease phenotype [33]. However, the moderate strength of this correlation suggests that BBB dysfunction represents one of multiple factors influencing disability progression in MS.

In this study, our results showed that age was significantly associated with EDSS score, which is consistent with the natural progression of multiple sclerosis. Indeed, increasing age has been identified as an important determinant of disability accumulation, reflecting both disease progression and neurodegenerative processes that may occur independently of inflammatory activity. Recent studies have demonstrated that older patients exhibit a steeper increase in EDSS scores over time and reach disability milestones more rapidly compared to younger individuals [34]. Furthermore, higher EDSS scores have been associated with advanced age and age-related comorbidities, suggesting that both disease-related and non-disease-related factors contribute to disability progression [35]. These findings support the role of age as a key factor influencing long-term disability in multiple sclerosis.

Interestingly, our study has indicated that the number of oligoclonal bands did not correlate with disability. This finding suggests that, while the presence of OCBs is important for diagnosis, their quantification does not necessarily reflect disease severity. Our results confirm the central role of intrathecal B-cell–mediated immunity in MS pathophysiology. The combination of a high IgG index, OCB positivity, and the Reiber classification provides a robust framework for assessing CNS immune activity. However, the relationship between these biomarkers and clinical outcomes remains complex and is likely influenced by other factors such as lesion burden, cortical involvement, and neurodegeneration.

One of the main findings of this study is the association between neurofilament levels and clinical disability. Indeed, patients with higher EDSS scores and progressive MS phenotypes increased neurofilament concentrations, supporting their role as a biomarker of axonal damage.

Neurofilaments are increasingly recognized as a sensitive marker of neuroaxonal damage in MS. Elevated levels have been associated with disease activity, progression, and long-term disability [36,37,38,39]. Our findings are consistent with these observations and suggest that neurofilament could reflect ongoing tissue damage with greater accuracy than conventional CSF markers.

pNF-H is a major cytoskeletal component of large myelinated axons, and its release into the CSF and, to a lesser extent, into the peripheral circulation reflects disruption of axonal integrity. Although the present study focused on multiple sclerosis, this mechanism is not disease-specific and may also occur in other neurological disorders characterized by acute or chronic axonal injury. In traumatic brain injury, axonal damage is part of a broader secondary injury cascade involving mitochondrial dysfunction, impaired bioenergetics, calcium dysregulation, oxidative stress, neuroinflammation, and delayed neurodegeneration. Himic et al. recently emphasized the theranostic relevance of mitochondrial dysfunction in traumatic brain injury and its contribution to secondary neuronal damage. In this context, our findings may have relevance beyond multiple sclerosis, as they support the broader value of pNF-H as a biomarker of neuroaxonal injury. Further studies comparing pNF-H dynamics across inflammatory demyelinating diseases and neurotrauma may help clarify its diagnostic, prognostic, and monitoring value in different mechanisms of central nervous system injury [40].

Importantly, although OCB and IgG index indicate immune activation, they do not directly measure neurodegeneration. The present data support the hypothesis that inflammation and neurodegeneration are partially dissociated processes in MS, with neurofilaments providing a more direct measure of irreversible tissue damage.

In this cohort, neurofilament levels increased in parallel with EDSS, supporting their role as a quantitative biomarker of disease severity. This observation is consistent with previous studies which have demonstrated that neurofilament levels are correlated with relapse activity, MRI lesion burden, brain atrophy, and long-term disability progression [41]. Moreover, the stronger association of neurofilaments with EDSS suggests that clinical disability is more related to neuroaxonal loss than to the extent of intrathecal immunoglobulin synthesis.

## 5. Conclusions

pNF-H concentrations in CSF appear to be a promising biomarker of axonal injury in Algerian patients with multiple sclerosis. In the present cross-sectional study, higher CSF pNF-H levels were associated with greater neurological disability, as reflected by EDSS scores, supporting their relationship with neuroaxonal damage. These findings are consistent with previous evidence suggesting that neurofilament proteins reflect neuronal and axonal injury in multiple sclerosis. Our results indicate that CSF pNF-H may provide complementary information to conventional CSF biomarkers by offering a quantitative measure of axonal damage. However, because of the cross-sectional design of this study, no conclusions can be drawn regarding its prognostic value, its ability to predict disease progression, or its usefulness for monitoring treatment response. Future longitudinal studies with larger cohorts and comprehensive clinical and MRI data are warranted to determine whether pNF-H can serve as a reliable biomarker for disease progression, therapeutic monitoring, and patient stratification in multiple sclerosis.

## 6. Limitations of the Study

This study has several limitations that should be acknowledged. First, the sample size was relatively limited, which may reduce the statistical power of some subgroup analyses and limit the generalizability of the findings. Second, this was a single-center study conducted in an Algerian cohort; therefore, validation in larger, multicenter and ethnically diverse populations is required. Third, the study design was cross-sectional, which does not allow assessment of longitudinal pNF-H dynamics over the course of disease progression, relapse, remission, or treatment response. Fourth, although pNF-H reflects axonal injury, it is not specific to multiple sclerosis and may be elevated in other neurological disorders involving neuroaxonal damage. Finally, serum pNF-H measurements may be influenced by peripheral clearance, blood– CSF barrier function, assay sensitivity, and disease stage. Future longitudinal studies integrating clinical disability scores, MRI measures, treatment status, and additional neurofilament biomarkers, particularly neurofilament light chain, would help clarify the prognostic and monitoring value of pNF-H in Algerian patients with multiple sclerosis.

## Figures and Tables

**Table 1:** Clinical characteristics of patients.

Variable	MS (*n* = 69)	NSDs (*n* = 33)
**Gender**		
Male, *n* (%)	31 (44.9%)	22 (66.6%)
Female, *n* (%)	38 (55.0%)	11 (33.3%)
Age (years)	30.0 [23.5–40.0]	43.5 [29.25–54.75]
**MS phenotype**		
RRMS, *n* (%)	53/69 (76.8%)	-
PMS, *n* (%)	16/69 (23.18%)	-
EDSS	2.0 [1.25–3.25]	-
**Oligoclonal bands (OCB)**		
OCB positive	59/69 (85.5%)	5/33 (15.15%)
OCB negative	10/69 (14.4%)	28/33 (84.84%)
OCB count	6 [4.5–9]	-
IgG index	0.87 [0.64–1.17]	0.52 [0.48–0.60]
IgG index > 0.7	47/69 (68.11%)	5/33 (15.15%)
Alb_Q	4.70 [3.25–6.11]	4.77 [4.15–7.01]
Intrathecal synthesis	45/69 (65.21%)	4/33 (12.12%)

**Table 2:** Correlation between EDSS and Clinical/Biochemical Parameters.

EDSS	Age	AlbQ	IgG Index	OCB
ρ	0.46	0.31	0.45	0.02
*p*	0.0007 ***	0.01 *	0.001 **	0.87 ^ns^

ns, not significant (*p* ≥ 0.05); * *p* < 0.05; ** *p* < 0.01; *** *p* < 0.001.

## Data Availability

The data presented in this study are available on request from the corresponding author.

## References

[1] Papiri G., D’Andreamatteo G., Cacchiò G., Alia S., Silvestrini M., Paci C., Luzzi S., Vignini A. (2023). Multiple Sclerosis: Inflammatory and Neuroglial Aspects. Curr. Issues Mol. Biol..

[2] Reich D.S., Lucchinetti C.F., Calabresi P.A. (2018). Multiple sclerosis. N. Engl. J. Med..

[3] Cencioni M.T., Villar L.M., Magliozzi R. (2024). Editorial: Community series in reassessing the immune system contribution in multiple sclerosis: Therapeutic target, biomarkers of disease and immune pathogenesis, volume II. Front. Immunol..

[4] Aburashed R., Eghzawi A., Long D., Pace R., Madha A., Cote J. (2025). Neurofilament light chain and multiple sclerosis: Building a neurofoundational model of biomarkers and diagnosis. Neurol. Int..

[5] Ebrahimi A., Wiil U.K., Olsson T., Liò P., Kockum I., Manouchehrinia A., Kiani N.A. (2026). A susceptibility network analysis of disease trajectories leading to multiple sclerosis: A nationwide cohort study. Mult. Scler..

[6] Petrova N., Carassiti D., Altmann D.R., Baker D., Schmierer K. (2018). Axonal loss in the multiple sclerosis spinal cord revisited. Brain Pathol..

[7] De Angelis F., Cameron J.R., Eshaghi A., Parker R., Connick P., Stutters J., Plantone D., Doshi A., John N., Williams T. (2025). Optical coherence tomography in secondary progressive multiple sclerosis: Cross-sectional and longitudinal exploratory analysis from the MS-SMART randomised controlled trial. J. Neurol. Neurosurg. Psychiatry.

[8] Garton T., Gadani S.P., Gill A.J., Calabresi P.A. (2024). Neurodegeneration and demyelination in multiple sclerosis. Neuron.

[9] Jakimovski D., Bittner S., Zivadinov R., Morrow S.A., Benedict R.H.B., Zipp F., Weinstock-Guttman B. (2024). Multiple sclerosis. Lancet.

[10] Maglio G., D’Agostino M., Caronte F.P., Pezone L., Casamassimi A., Rienzo M., Di Zazzo E., Nappo C., Medici N., Molinari A.M. (2024). Multiple sclerosis: From the application of oligoclonal bands to novel potential biomarkers. Int. J. Mol. Sci..

[11] Molligoda Arachchige A.S.P., El Choueiri J., Pellicanò F., Laurelli F., Alves G.A.M., Stomeo N. (2025). A review of multiple sclerosis: From pathophysiology to latest therapeutic advances. AIMS Neurosci..

[12] Boutitah-Benyaich I., Eixarch H., Villacieros-Álvarez J., Hervera A., Cobo-Calvo Á., Montalban X., Espejo C. (2025). Multiple sclerosis: Molecular pathogenesis and therapeutic intervention. Signal Transduct. Target. Ther..

[13] Monreal E., Fernández-Velasco J.I., Álvarez-Lafuente R., Sainz de la Maza S., García-Sánchez M.I., Llufriu S., Casanova B., Comabella M., Martínez-Yélamos S., Galimberti D. (2024). Serum biomarkers at disease onset for personalized therapy in multiple sclerosis. Brain.

[14] Zucchi E., Bonetto V., Sorarù G., Martinelli I., Parchi P., Liguori R., Mandrioli J. (2020). Neurofilaments in motor neuron disorders: Towards promising diagnostic and prognostic biomarkers. Mol. Neurodegener..

[15] Biernacki T., Kokas Z., Sandi D., Füvesi J., Fricska-Nagy Z., Faragó P., Kincses T.Z., Klivényi P., Bencsik K., Vécsei L. (2022). Emerging biomarkers of multiple sclerosis in the blood and the CSF: A focus on neurofilaments and therapeutic considerations. Int. J. Mol. Sci..

[16] van Asperen J.V., Kotaich F., Caillol D., Bomont P. (2024). Neurofilaments: Novel findings and future challenges. Curr. Opin. Cell Biol..

[17] van Zeggeren I.E., Ter Horst L., Heijst H., Teunissen C.E., van de Beek D., Brouwer M.C. (2022). Neurofilament light chain in central nervous system infections: A prospective study of diagnostic accuracy. Sci. Rep..

[18] Liampas I., Kyriakoulopoulou P., Karakoida V., Kavvoura P.A., Sgantzos M., Bogdanos D.P., Stamati P., Dardiotis E., Siokas V. (2024). Blood-based biomarkers in frontotemporal dementia: A narrative review. Int. J. Mol. Sci..

[19] Gallingani C., Carbone C., Tondelli M., Zamboni G. (2024). Neurofilament light chain in neurodegenerative dementias: A review of imaging correlates. Brain Sci..

[20] Demiri S., Veltsista D., Siokas V., Spiliopoulos K.C., Tsika A., Stamati P., Chroni E., Dardiotis E., Liampas I. (2025). Neurofilament light chain in cerebrospinal fluid and blood in multiple system atrophy: A systematic review and meta-analysis. Brain Sci..

[21] Pavelek Z., Souček O., Krejsek J., Součková I., Popovičová A., Matyáš D., Sobíšek L., Novotný M. (2025). Exploring the prognostic role of neurofilaments and SEMA3A in multiple sclerosis progression. Int. J. Mol. Sci..

[22] Yuan A., Rao M.V., Veeranna N., Nixon R.A. (2017). Neurofilaments and neurofilament proteins in health and disease. Cold Spring Harb. Perspect. Biol..

[23] Gafson A.R., Barthélemy N.R., Bomont P., Carare R.O., Durham H.D., Julien J.-P., Kuhle J., Leppert D., Nixon R.A., Weller R.O. (2020). Neurofilaments: Neurobiological foundations for biomarker applications. Brain.

[24] Bittner S., Oh J., Havrdová E.K., Tintoré M., Zipp F. (2021). The potential of serum neurofilament as biomarker for multiple sclerosis. Brain.

[25] Devarakonda S.S., Basha S., Pithakumar A., Mukunda D.C., Rodrigues J., Biswas S., Pai A.R., Belurkar S., Mahato K.K. (2024). Molecular mechanisms of neurofilament alterations and its application in assessing neurodegenerative disorders. Ageing Res. Rev..

[26] Ciubotaru A., Smihor M.I., Grosu C., Alexa D., Covali R., Anicăi R.-C., Păvăleanu I., Cucu A.I., Bobu A.M., Ghiciuc C.M. (2025). Neurodegenerative biomarkers in multiple sclerosis: At the interface between research and clinical practice. Diagnostics.

[27] Daponte A., Koros C., Skarlis C., Siozios D., Rentzos M., Papageorgiou S.G., Anagnostouli M. (2025). Neurofilament biomarkers in neurology: From neuroinflammation to neurodegeneration, bridging established and novel analytical advances with clinical practice. Int. J. Mol. Sci..

[28] Thompson A.J., Banwell B.L., Barkhof F., Carroll W.M., Coetzee T., Comi G., Correale J., Fazekas F., Filippi M., Freedman M.S. (2018). Diagnosis of multiple sclerosis: 2017 revisions of the McDonald criteria. Lancet Neurol..

[29] Dobson R., Giovannoni G. (2019). Multiple sclerosis—A review. Eur. J. Neurol..

[30] Reiber H., Peter J.B. (2001). Cerebrospinal fluid analysis: Disease-related data patterns and evaluation programs. J. Neurol. Sci..

[31] Vlad B., Hilty M., Neidhart S., Asplund Högelin K., Ziegler M., Khademi M., Lutterotti A., Regeniter A., Martin R., Al Nimer F. (2024). Intensity of intrathecal total IgG synthesis in multiple sclerosis correlates with the degree of pleocytosis, diversity of intrathecal antiviral antibody specificities, and female sex. Antibodies.

[32] Rodriguez-Mogeda C., van Gool M.M.J., van der Mast R., Nijland R., Keasberry Z., van de Bovekamp L., van Delft M.A.M., Picon C., Reynolds R., Killestein J. (2024). Intrathecal IgG and IgM synthesis correlates with neurodegeneration markers and corresponds to meningeal B cell presence in MS. Sci. Rep..

[33] Puthenparampil M., Marin A., Zanotelli G., Mauceri V.A., De Napoli F., Gaggiola M., Miscioscia A., Ponzano M., Bovis F., Perini P. (2024). Blood–brain barrier damage associates with glia-related cytokines in the cerebrospinal fluid of patients with multiple sclerosis. Mult. Scler. Relat. Disord..

[34] Simone M., Lucisano G., Guerra T., Paolicelli D., Rocca M.A., Brescia Morra V., Patti F., Annovazzi P., Gasperini C., De Luca G. (2024). Disability trajectories by progression independent of relapse activity status differ in pediatric, adult and late-onset multiple sclerosis. J. Neurol..

[35] Lynch S., Baker S., Nashatizadeh M., Thuringer A., Thelen J., Bruce J. (2021). Disability measurement in multiple sclerosis patients 55 years and older: What is the Expanded Disability Status Scale really telling clinicians?. Mult. Scler. Relat. Disord..

[36] Disanto G., Barro C., Benkert P., Naegelin Y., Schädelin S., Giardiello A., Zecca C., Blennow K., Zetterberg H., Leppert D. (2017). Serum neurofilament light: A biomarker of neuronal damage in multiple sclerosis. Ann. Neurol..

[37] Konitsioti A.M., Göreci Y., Hellmich M., Schweitzer F., Blaschke S., Golla H., Waßner L., Stolzenberger J., Fink G.R., Schroeter M. (2026). Impact of whole-body vibration training on gait in patients with progressive multiple sclerosis: A pilot randomised study. BMJ Neurol. Open.

[38] García Domínguez J.M., Villar L.M., Arrambide G., Blanco Y., Calles M.C., Casanova B., Castillo-Triviño T., Costa-Frossard França L., Eichau S., Hernández Pérez M.Á. (2025). Relevance of serum neurofilament determination as a biomarker in multiple sclerosis. Neurology.

[39] Chitnis T., Magliozzi R., Abdelhak A., Kuhle J., Leppert D., Bielekova B. (2025). Blood and CSF biomarkers for multiple sclerosis: Emerging clinical applications. Lancet Neurol..

[40] Himic V., Tchantchaleishvili N., Netliukh A., Chibbaro S., Syrmos N., Ligarotti G.K.I., Prisco L., Ganau M. (2026). Mitochondrial Dysfunction in Traumatic Brain Injury and Its Theranostic Implications. Biomolecules.

[41] Dimitriadou E.M., Tzanetakos D., Theodorou A., Akrivaki A., Papadopoulos D., Mastorodemos V., Maleska A., Sotirli S.A., Fakas N., Stergiou C. (2026). The predictive value of serum neurofilament light chain in achieving no evidence of disease activity-3 (NEDA-3) status in treatment-naïve patients with multiple sclerosis. J. Neurol..

